# Supplementary material for: Predicting Immunotherapy Efficacy with Machine Learning in Gastrointestinal Cancers: A Systematic Review and Meta-Analysis
Source: Int J Mol Sci. 2025 Jun 20;26(13):5937. doi: 10.3390/ijms26135937 (PMC12250185; doi:10.3390/ijms26135937)
Supplement: Supplementary file 1 [file ijms-26-05937-s001.zip › Supplementary File S1.pdf]

Short:

(immunotherapy OR ICI OR immune checkpoint) AND (machine learning OR deep learning OR artificial intelligence) AND (cancer OR tumor) AND (survival OR prognosis OR response OR predict)
+
("ai"[Title/Abstract] OR "ai"[Text Word] OR "ai"[Other Term])

Total translation:

("immunotherapy"[MeSH Terms] OR "immunotherapy"[All Fields] OR "immunotherapies"[All Fields] OR "immunotherapy s"[All Fields] OR "ICI"[All Fields] OR (("immune"[All Fields] OR "immuned"[All Fields] OR "immunes"[All Fields] OR "immunisation"[All Fields] OR "vaccination"[MeSH Terms] OR "vaccination"[All Fields] OR "immunization"[All Fields] OR "immunization"[MeSH Terms] OR "immunisations"[All Fields] OR "immunizations"[All Fields] OR "immunise"[All Fields] OR "immunised"[All Fields] OR "immuniser"[All Fields] OR "immunisers"[All Fields] OR "immunising"[All Fields] OR "immunities"[All Fields] OR "immunity"[MeSH Terms] OR "immunity"[All Fields] OR "immunization s"[All Fields] OR "immunize"[All Fields] OR "immunized"[All Fields] OR "immunizer"[All Fields] OR "immunizers"[All Fields] OR "immunizes"[All Fields] OR "immunizing"[All Fields]) AND ("cell cycle checkpoints"[MeSH Terms] OR ("cell"[All Fields] **AND** "cycle"[All Fields] AND "checkpoints"[All Fields]) OR "cell cycle checkpoints"[All Fields] OR "checkpoint"[All Fields] OR "checkpoints"[All Fields]))) **AND** ("machine learning"[MeSH Terms] OR ("machine"[All Fields] **AND** "learning"[All Fields]) OR "machine learning"[All Fields] OR ("deep learning"[MeSH Terms] OR ("deep"[All Fields] AND "learning"[All Fields]) OR "deep learning"[All Fields]) OR ("artificial intelligence"[MeSH Terms] OR ("artificial"[All Fields] **AND** "intelligence"[All Fields]) OR "artificial intelligence"[All Fields]) OR ("ai"[Title/Abstract] OR "ai"[Text Word] OR "ai"[Other Term])) **AND** ("cancer s"[All Fields] OR "cancerated"[All Fields] OR "canceration"[All Fields] OR "cancerization"[All Fields] OR "cancerized"[All Fields] OR "cancerous"[All Fields] OR "neoplasms"[MeSH Terms] OR "neoplasms"[All Fields] OR "cancer"[All Fields] OR "cancers"[All Fields] OR ("cysts"[MeSH Terms] OR "cysts"[All Fields] OR "cyst"[All Fields] OR "neurofibroma"[MeSH Terms] OR "neurofibroma"[All Fields] OR "neurofibromas"[All Fields] OR "tumor s"[All Fields] OR "tumoral"[All Fields] OR "tumorous"[All Fields] OR "tumour"[All Fields] OR "neoplasms"[MeSH Terms] OR "neoplasms"[All Fields] OR "tumor"[All Fields] OR "tumour s"[All Fields] OR "tumoural"[All Fields] OR "tumourous"[All Fields] OR "tumours"[All Fields] OR "tumors"[All Fields])) **AND** ("mortality"[MeSH Subheading] OR "mortality"[All Fields] OR "survival"[All Fields] OR "survival"[MeSH Terms] OR "survivability"[All Fields] OR "survivable"[All Fields] OR "survivals"[All Fields] OR "survive"[All Fields] OR "survived"[All Fields] OR "survives"[All Fields] OR "surviving"[All Fields] OR ("prognosis"[MeSH Terms] OR "prognosis"[All Fields] OR "prognoses"[All Fields]) OR ("response"[All Fields] OR "responses"[All Fields] OR "responsive"[All Fields] OR "responsiveness"[All Fields] OR "responsivenesses"[All Fields] OR "responsives"[All Fields] OR "responsivities"[All Fields] OR "responsivity"[All Fields]) OR ("predict"[All Fields] OR "predictabilities"[All Fields] OR "predictability"[All Fields] OR "predictable"[All Fields] OR "predictably"[All Fields] OR "predicted"[All Fields] OR "predicting"[All Fields] OR "prediction"[All Fields] OR "predictions"[All Fields] OR "predictive"[All Fields] OR "predictively"[All Fields] OR "predictiveness"[All Fields] OR "predictives"[All Fields] OR "predictivities"[All Fields] OR "predictivity"[All Fields] OR "predicts"[All Fields]))
