# Supplementary material for: Predicting Immunotherapy Efficacy with Machine Learning in Gastrointestinal Cancers: A Systematic Review and Meta-Analysis
Source: Int J Mol Sci. 2025 Jun 20;26(13):5937. doi: 10.3390/ijms26135937 (PMC12250185; doi:10.3390/ijms26135937)
Supplement: Supplementary file 1 [file ijms-26-05937-s001.zip › Table S1.pdf]

Table S1. NEWCASTLE-OTTAWA scale for cohort studies

Study	Year	Selection	Comparability	Outcome
Liu et al.	2022.0	★★	★★	★★
Lu et al.	2020.0	★★	★★	★★
Cheong et al.	2022.0	★★	★★	★★★
Wei et al.	2022.0	★★	★★	★★
He et al.	2022.0	★★★	★★	★★
Tang et al.	2022.0	★★★	★★	★★
Zhou et al.	2021.0	★★	★★	★★
Lee et al.	2021.0	★★	★★	★★
Zhao et al.	2023.0	★★	★★	★★
