# Supplementary figures and images for: Predicting Immunotherapy Efficacy with Machine Learning in Gastrointestinal Cancers: A Systematic Review and Meta-Analysis
Source: Int J Mol Sci. 2025 Jun 20;26(13):5937. doi: 10.3390/ijms26135937 (PMC12250185; doi:10.3390/ijms26135937)

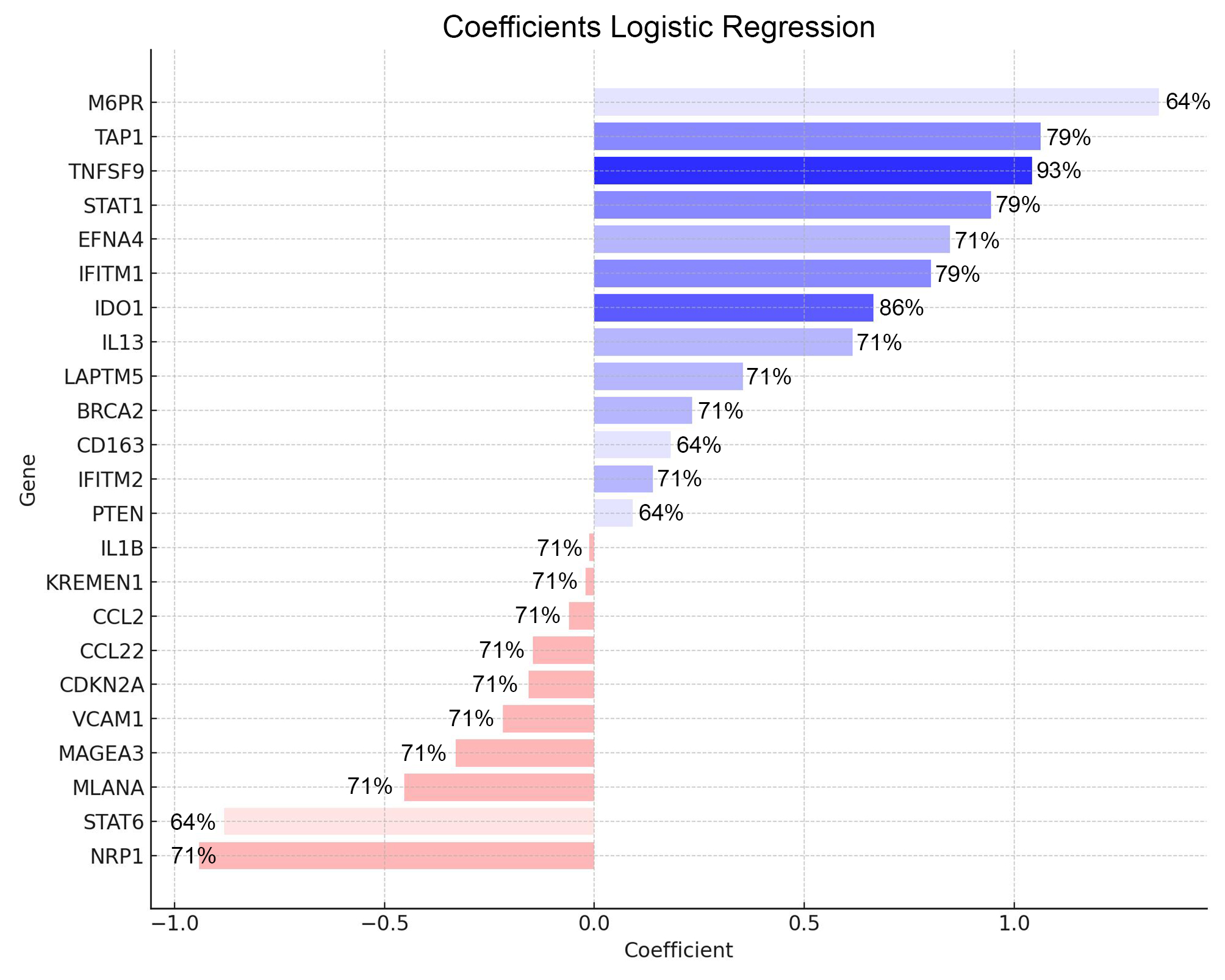

Supplement: Supplementary file 1 [file ijms-26-05937-s001.zip › Figure S1.jpeg]

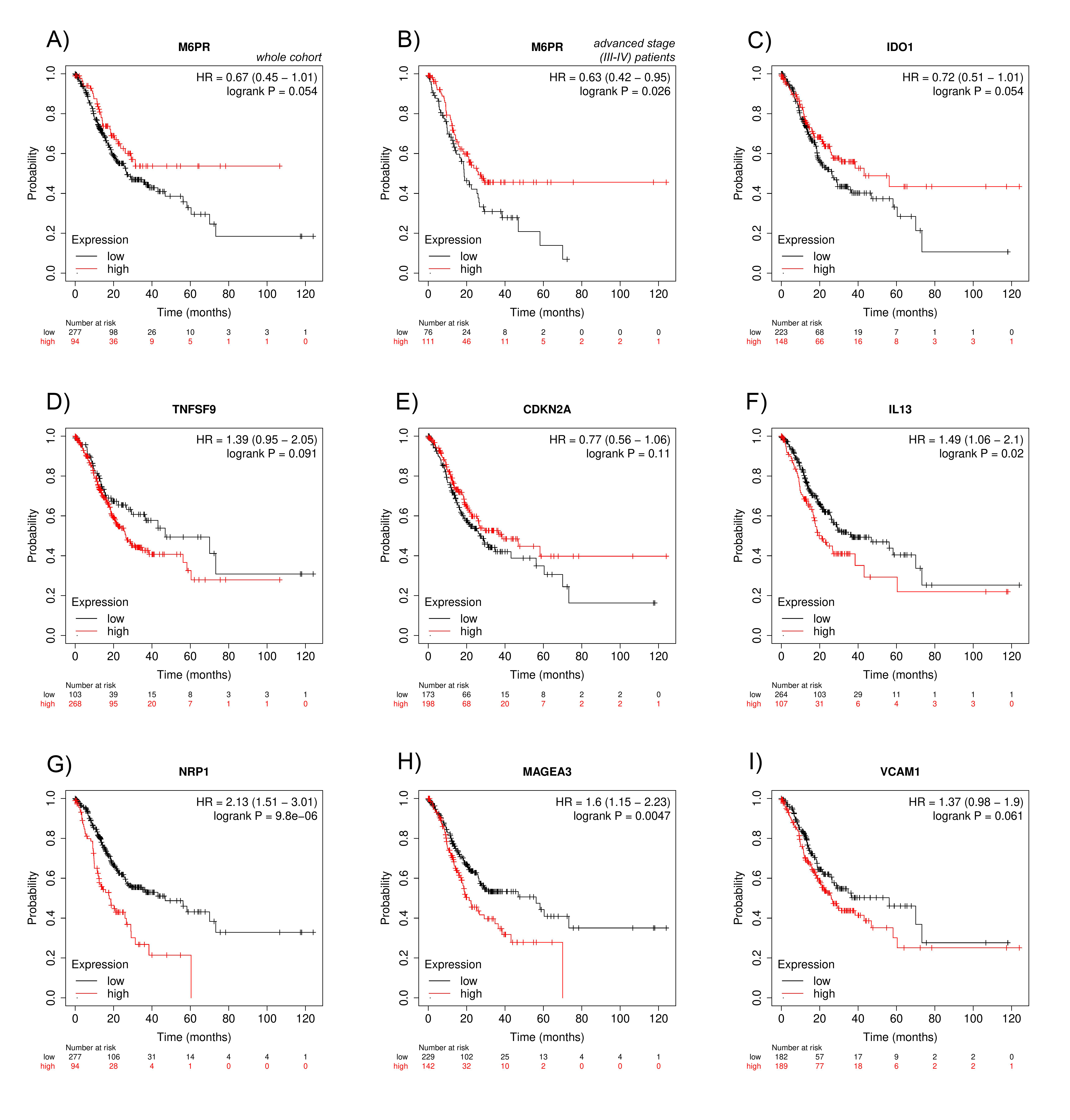

Supplement: Supplementary file 1 [file ijms-26-05937-s001.zip › Figure S2.jpg]
